# Clinical Characteristics and Lesions Responsible for Swallowing Hesitation After Acute Cerebral Infarction

**DOI:** 10.1007/s00455-016-9716-8

**Published:** 2016-06-08

**Authors:** Tsukasa Saito, Keisuke Hayashi, Hajime Nakazawa, Tetsuo Ota

**Affiliations:** Department of Physical Medicine and Rehabilitation, Asahikawa Medical University Hospital, Midorigaoka Higashi 2-1-1-1, Asahikawa, Hokkaido 078-8510 Japan; Cardiovascular, Respiratory and Neurology Division, Department of Internal Medicine, Asahikawa Medical University, Asahikawa, Japan

**Keywords:** Dysphagia, Swallowing, Deglutition, Videofluorography, Hesitation, Deglutition disorders

## Abstract

Some stroke patients with a unilateral lesion demonstrate acute dysphagia characterized by a markedly prolonged swallowing time, making us think they are reluctant to swallow. In order to clarify the clinical characteristics and causative lesions of delayed swallowing, we conducted a retrospective analysis of 20 right-handed patients without a history of swallowing dysfunction who underwent videofluorography on suspicion of dysphagia after a first ischemic stroke. The oral processing time plus the postfaucial aggregation time required to swallow jelly for patients classified as having delayed swallowing was over 10 s. The time required for swallowing jelly was significantly longer than that without the hesitation (median value, 24.1 vs. 8.9 s, *P* < 0.001). The oral processing time plus the postfaucial aggregation time required for patients with delayed swallowing to swallow thickened water was largely over 5 s and significantly longer than that of patients without swallowing hesitation (median value, 10.2 vs. 3.3 s, *P* < 0.001). Swallowing hesitation caused by acute unilateral infarction could be separated into two different patterns. Because four of the five patients with a rippling tongue movement in the swallowing hesitation pattern had a lesion in the left primary motor cortex, which induces some kinds of apraxia, swallowing hesitation with a rippling tongue movement seems to be a representative characteristic of apraxia. The patients with swallowing hesitation with a temporary stasis of the tongue in this study tended to have broad lesions in the frontal lobe, especially in the middle frontal gyrus, which is thought to be involved in higher cognition.

**Electronic supplementary material:**

The online version of this article (doi:10.1007/s00455-016-9716-8) contains supplementary material, which is available to authorized users.

## Introduction

Stroke is one of the most common causes of dysphagia [1]. Dysphagia not only carries the possibility of fatal aspiration pneumonia but also undermines the patient’s quality of life. Therefore, early detection of dysphagia and quick induction of rehabilitation is very important. Videofluorography (VF) is considered as the gold standard for evaluation and management of dysphagia [2–4]. Even in the acute phase of a stroke, VF is widely practiced as a safe and potent procedure for diagnosis of dysphagia.

Dysphagia in acute cerebrovascular disease is generally due to synchronized multiple infarcts affecting the bilateral corticobulbar tracts or unilateral ventral bulbar involvement. In other words, dysphagia in stroke is considered a subcategory of pseudobulbar or bulbar palsy. However, we sometimes encounter acute dysphagia in stroke patients, and we inevitably conclude that unilateral lesions cause the dysphagia [5, 6]. Further, we sometimes notice that this type of acute dysphagia is characterized by a markedly prolonged swallowing time. Once the deglutition reflex starts, almost all of these patients can swallow water or a bolus easily without penetration or aspiration, but requiring a long time to swallow suggests that they are hesitating to swallow the water or bolus.

Deglutition is a fragile mechanism comprising the precise cooperation of many organs and the central nervous system. Despite many previous studies, the details of sensorimotor integration in swallowing remain unknown, and comprehensive models for neural control of swallowing have not been well defined. Similarly, causative lesions and characteristics of swallowing hesitation in acute cerebrovascular disease are yet unclear. We hypothesized that swallowing hesitation, seemingly a mere embarrassment in swallowing, is a syndrome of dysphagia that truly requires significantly extended time to swallow and contains more than one specific characteristic. Further, we also hypothesized that a left frontal lesion including the primary motor cortex could cause significant swallowing hesitation. In order to prove these hypotheses, in other words to clarify the clinical features and responsible lesions of patients with swallowing hesitation, we conducted a retrospective study of consecutive acute stroke patients with imaging and neuropsychology in detail.

## Methods

The entire study protocol was approved by the ethical review board of Asahikawa Medical University, and all patients gave their written informed consent for the present study.

### Patient Enrollment

We conducted a retrospective review of the medical records of 207 consecutive acute ischemic stroke patients admitted to Asahikawa Medical University Hospital from September 2014 to December 2015. Of these, we enrolled 20 patients who underwent VF on suspicion of dysphagia with the first ischemic stroke and without a history of swallowing dysfunction. The diagnosis of acute ischemic stroke was based on neurological examination by certified stroke specialists and magnetic resonance imaging (MRI). A brain computed tomography (CT) scan was also performed on patients with an implanted pacemaker. The retrospective CT and MRI assessments were performed by trained observers (T.S. and T.O.) who were blinded to the VF information. A cerebral infarction was defined as a hyperintense lesion in the diffusion-weighted image (DWI), with a corresponding hyperintensity on the fluid-attenuated inversion recovery (FLAIR) sequence images and T2-weighted images (T2WI) in the subacute or chronic phase. Moreover, we excluded patients with a contralateral white matter abnormality which was recognized as a hyperintense area in the FLAIR and T2WI or low-density areas on brain CT in the acute phase. Accordingly, patients with acute bilateral lesions or diffuse white matter abnormality were excluded.

### Assessment of VF

The VF assessments were performed by trained speech-language-hearing therapists (K.H. and H.N.) using a standard protocol [7–9] within 4 weeks of admission. A video recording of the oral cavity and pharynx was obtained in the lateral plane while the patient swallowed sequentially 3 ml of water and then one teaspoonful of jelly, both blended with barium as a contrast material. The barium solution in water (50 % weight/volume ratio) was thickened with 0.5 % dextrin. We made a barium jelly of normal consistency from the aforementioned barium water with 2 % gelatin.

We repeatedly instructed the patients by gesticulation to swallow the water or jelly as quickly as possible, and ensured the patients’ understanding by their distinct nods of approval. We injected water into the mouth using a syringe and jelly into their mouth using a spoon. After we asked the patients to open his or her mouth naturally, we put the jelly on the center of the tongue with a teaspoon. Then, we told the patients to close their mouth, and we quickly pulled the teaspoon out of their mouth from the patients’ lips. We told the patients to swallow immediately after injection. We did not recommend chewing.

Retrospectively, we measured the time from the moment water or jelly first entered the oral cavity until the leading edge of the contrast material reached the inferior border of the mandible correctly. The time required for patients to swallow corresponds to the oral processing time (OPT) plus the postfaucial aggregation time (PFAT) [10, 11]. We divided swallowing hesitations into two groups according to the characteristics of the transfer dysfunction. The “stasis” group produced an oral phase characterized by a lack of movement of the tongue and consequent stagnation of water or jelly on the floor of the oral cavity. On the other hand, the “rippling” group presented an oral phase characterized by a repetitive rippling tongue movement.

### Statistical Analysis

Statistical analyses were performed using SPSS version 11.0 software (SPSS Inc., Chicago, IL, USA). We statistically compared OPT + PFAT of each group as a dependent variable. Student’s *t* test was used for the group comparisons of age. The OPT + PFAT were compared using the Mann–Whitney *U* test. We considered *P* < 0.05 as statistically significant.

## Results

Twenty patients with acute cerebral infarction were enrolled in the study (Table 1). None of the eligible patients exhibited paralysis of the tongue or palate; in other words, they did not have bulbar palsy. None of them were deaf, had consciousness disturbance, or severe cognitive dysfunction. All 20 patients were right-handed and had no old infarction or significant white matter abnormality on the contralateral side. Ten of the twenty patients required more than 10 s for OPT + PFAT with jelly and were classified as having swallowing hesitation. These ten patients with swallowing hesitation consisted of nine patients with new lesions of the left cerebral hemisphere and one in the right cerebral hemisphere. Nine of the ten patients with swallowing hesitation showed aphasia. The patients in the swallowing hesitation group with apraxia showed hand dyspraxia as well. The mean ages of the two groups did not differ significantly (with swallowing hesitation = 77.3 ± 8.6 years vs. without swallowing hesitation = 75.4 ± 12.3 years).

**Table 1:** Clinical profiles of 20 patients and each detail site of lesions

No.	Age (years)	Sex	Days	Hesitation	Aphasia	Apraxia	Lesions	OFC	SFG	MFG
1	84	F	6	Rippling	+	+	Lt hemisphere	−	−	−
2	75	F	10	Rippling	+	+	Lt hemisphere	−	−	−
3	73	F	8	Rippling	+	+	Lt hemisphere	−	−	−
4	79	M	6	Rippling	−	−	Rt hemisphere	−	−	−
5	80	F	6	Rippling	+	+	Lt hemisphere	−	−	−
6	91	F	5	Stasis	+	−	Lt hemisphere	−	−	−
7	85	F	7	Stasis	+	+	Lt hemisphere	−	−	+
8	66	F	22	Stasis	+	+	Lt hemisphere	+	+	+
9	63	M	37	Stasis	+	+	Lt hemisphere	+	−	+
10	77	F	27	Stasis	+	+	Lt hemisphere	+	−	+
11	79	F	7	−	−	−	Lt hemisphere	−	−	−
12	71	M	5	−	−	−	Rt hemisphere	−	−	−
13	46	F	14	−	−	−	Lt hemisphere	−	−	−
14	88	M	16	−	−	−	Rt hemisphere	−	−	−
15	72	F	39	−	−	−	Rt hemisphere	−	−	+
16	75	M	17	−	−	−	Rt cerebellum	−	−	−
17	72	M	10	−	+	+	Lt hemisphere	+	−	−
18	76	M	7	−	−	−	Rt hemisphere	−	−	−
19	88	F	6	−	−	−	Rt hemisphere	−	−	−
20	87	F	13	−	−	−	Rt hemisphere	−	−	−

No.	IFG	Insula	SMA	PMC	PSC	AG	Cap/CR	Water (s)	Jelly (s)
1	−	−	−	+	−	−	−	6.4	12.0
2	−	+	−	+	+	+	−	5.7	25.0
3	−	+	−	+	−	+	+	9.6	16.4
4	−	−	−	+	−	−	−	4.8	23.1
5	−	+	−	−	+	−	−	28.3	24.0
6	−	+	−	−	−	−	−	9.5	99.6
7	−	−	−	+	−	−	−	17.9	120.0
8	+	+	+	+	+	+	−	31.8	24.2
9	+	+	−	+	+	+	+	10.7	51.0
10	+	+	−	+	−	−	+	25.2	16.1
11	−	−	−	−	−	−	+	3.0	6.9
12	−	+	−	+	+	+	−	2.6	6.1
13	−	−	−	−	−	−	+	1.2	4.5
14	+	+	−	+	+	−	−	5.1	10.2
15	+	−	−	−	−	−	−	9.4	10.7
16	−	−	−	−	−	−	−	2.4	10.3
17	+	+	−	−	−	−	−	3.6	7.3
18	−	−	−	−	−	−	+	3.7	10.5
19	−	−	−	−	−	−	+	2.6	8.5
20	−	−	−	−	−	−	+	5.0	9.3

Note that patients 1–10 showed swallowing hesitation, and 11–20 did not

*Days* the number of days from onset to videofluorography, *OFC* orbitofrontal cortex, *SFG* superior frontal gyrus, *MFG* middle frontal gyrus, *IFG* inferior frontal gyrus, *SMA* supplementary motor area, *PMC* primary motor cortex, *PSC* primary sensory cortex, *AG* angular gyrus, *cap*/*CR* capsule/corona radiata, *F* female, *M* male, *lt* left, *rt* right

The OPT + PFAT required for patients with swallowing hesitation to swallow the thickened water was largely over 5 s. The OPT + PFAT of swallowing hesitation was significantly longer than that of patients without swallowing hesitation (median value, 10.2 vs. 3.3 s, *P* < 0.001). As mentioned above, the OPT + PFAT required for patients with swallowing hesitation to swallow the jelly was all over 10 s and gave an impression of hesitation. The OPT + PFAT required for patients with swallowing hesitation to swallow jelly was significantly longer than that of patients without hesitation (median value, 24.1 vs. 8.9 s, *P* < 0.001).

Both groups took a long time to swallow. The OPT + PFAT required for patients with ripplet swallowing hesitation to swallow water was not significantly longer than that of patients with stasis swallowing hesitation (median value, 6.4 vs. 17.9 s, *P* = 0.12). The same was true for jelly (median value, 23.1 s of rippling group vs. 51.0 s of stasis group, *P* = 0.12). However, the two groups varied in the distribution of lesions. Four of the five patients of the rippling group displayed limited ischemia, particularly in the primary motor cortex. Further, four of the five patients in the stasis group had a larger lesion in the frontal lobe, including the middle frontal gyrus and primary motor cortex. Liberally interpreted, the orbitofrontal cortex, middle and inferior frontal gyrus, insula, and primary motor cortex were involved with a high probability. We briefly present three representative cases below in order to elucidate the relationship between symptoms and responsible lesions.

### Representative Cases Presentation

#### Case 1 (Patient 1)

**Fig. 1:**
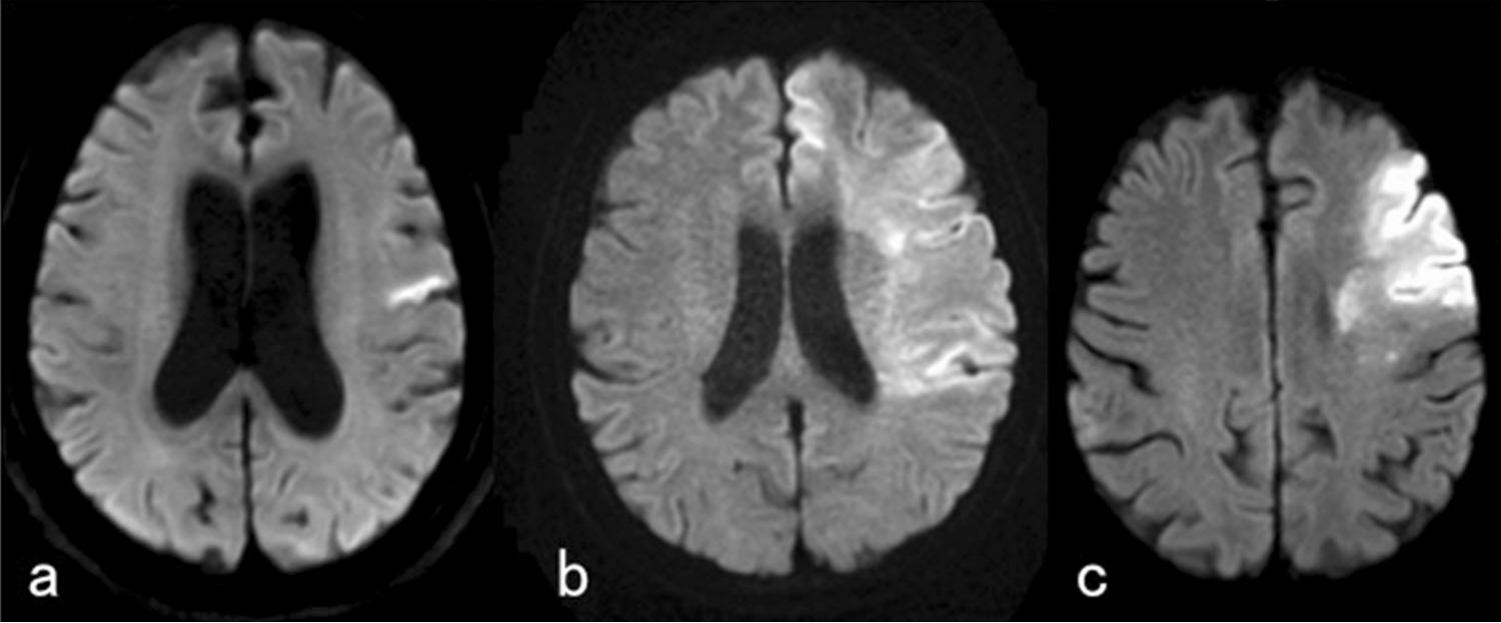
Diffusion-weighted magnetic resonance imaging (DWI) of three representative cases. **a** Case 1 demonstrated a left hemisphere lesion including the entire frontal lobe, primary sensory cortex, angular gyrus, and insula. **b** Case 2 suffered an extended left hemisphere lesion including the entire frontal lobe, primary sensory cortex, angular gyrus, and insula. **c** Case 3 had a relatively broad infarction including orbitofrontal cortex, inferior frontal gyrus, and insula of the left cerebral hemisphere; however, large parts of the middle frontal gyrus and primary motor cortex were spared

An 84-year-old woman was found lying on the floor with impaired awareness and transported to our hospital by ambulance. At presentation, the neurological examination by a stroke specialist demonstrated slight right-side upper and lower limb palsy, buccofacial apraxia, and motor aphasia. Palatoplegia or deviation of the tongue was not obvious. Sensory disturbance, including of the face and oral cavity, was not obvious. We did not perform a mental test in detail because of motor aphasia. Diffusion-weighted MRI revealed a new localized lesion in the left primary motor cortex (Fig. 1a). On the sixth day after onset, VF demonstrated swallowing hesitation with a typical repetitive rippling movement of the tongue (Video 1). The OPT + PFAT was 6.4 s for water and 12.0 s for jelly. From the day after VF, she started to eat softened food for dysphagia. She maintained adequate oral intake and good swallowing function while in our hospital without any obvious aspiration pneumonia. On the 20th day from onset, she left our hospital and was admitted to another hospital for further rehabilitation.

#### Case 2 (Patient 8)

A 66-year-old woman who fell suddenly while preparing lunch was taken to an emergency medical center of our hospital by ambulance. At presentation, the neurological examination by a stroke specialist demonstrated complete right-side palsy, apraxia of the right upper extremity, complete right-side anesthesia including the face, and total aphasia. However, sensory disturbance of the oral cavity was not examined. Total aphasia prevented us from evaluating the mental state. Although she displayed right-side facial palsy too, movement of the tongue and palate was not disturbed. Diffusion-weighted MRI revealed an extended left hemisphere lesion including the entire frontal lobe, primary sensory cortex, angular gyrus, and insula (Fig. 1b). On the 22nd day from onset, VF demonstrated swallowing hesitation with a typical pause pattern (Video 2). The OPT + PFAT was 31.8 s for water and 24.2 s for jelly. Despite the extended swallowing time on VF, she took adequate softened food by mouth with assistance. On the 41st day after onset, she was discharged from our hospital to continue further rehabilitation in another hospital.

#### Case 3 (Patient 17)

A 72-year-old man who suffered from dysarthria on awakening was transferred to our hospital by ambulance. At presentation in the emergency room, the neurological examination by a stroke specialist demonstrated severe buccofacial apraxia, ideomotor apraxia, marked dyspraxia of the right upper extremity, and total aphasia. Sensory disturbance of the right side of the body, including oral cavity and face, was not apparent. We could not evaluate his mental state due to his total aphasia. No motor palsy was obvious, including the tongue and palate. Diffusion-weighted MRI revealed a relatively broad infarction including the orbitofrontal cortex, inferior frontal gyrus, and insula in the left cerebral hemisphere; however, a large part of the middle frontal gyrus and primary motor cortex was spared (Fig. 1c). He started feeding by himself on the third day after onset. On the 10th day after onset, VF did not detect any swallowing hesitation. On the 17th day after onset, he left our hospital and went home.

## Discussion

This is the first report to describe two different clinical patterns of swallowing hesitation caused by an acute unilateral lesion, but it has potential use in many clinical cases. The caretakers feel that the patients are hesitating to swallow because of the longer time taken by them to swallow completely. Specifically, the time needed to swallow water is 5 s longer and to swallow jelly is about 10 s longer than normal. Although we had been unaware of its presence, swallowing hesitation may be considered as one of the dominant patterns of dysphagia characterized by a unique neurological deficiency.

### Corticobulbar Tract and Swallowing Hesitation

Nine of the ten patients with swallowing hesitation had a left hemisphere lesion. Interestingly, a few patients with suspicion of dysphagia in our study had a lesion in the left or right capsule or corona radiata. Deglutition and articulatory movements share a common anatomical structure and neurological basis. Some studies demonstrated that dysarthria after a single acute cerebral infarction in the area of the internal capsule or corona radiata is caused significantly and more frequently by a left-side lesion [12, 13]. The corticobulbar tract consists of two groups of fibers, crossing fibers that affect contralateral nuclei and uncrossing fibers that affect ipsilateral nuclei. In other words, nuclei of the medulla are controlled bilaterally by corticobulbar tracts. Therefore, a lesion of a unilateral corticobulbar tract does not ordinarily cause dysphagia. Further, the ratio of the two types of fibers varies among individuals [14]. When a patient has more crossing fibers and fewer uncrossing fibers bilaterally, a unilateral lesion may result in dysphagia. However, the entire picture of the human innate innervation and re-innervation has not been sufficiently revealed. The fact that hesitation does not originate in the motor cortex cannot be denied. Anyway, in this study, a lesion of the internal capsule or corona radiata seemed to have no relationship with swallowing hesitation. Further accumulation of cases and careful longitudinal studies are needed to clarify the relationship between corticobulbar tract and swallowing hesitation.

### Left Primary Motor Cortex and “Rippling” Swallowing Hesitation

Four of the five patients with the rippling swallowing hesitation pattern had a lesion in the left primary motor cortex. Moreover, Patient 1 (Case 1) and four who presented rippling tongue movements on VF had a lesion only in the primary motor cortex. The rippling tongue movement appears to be a repetition of trial and error in deglutition. Although the actual motor functions of muscles involved in deglutition are intact, a lack of rhythmical and disciplined coordination of them results in hesitation. The lesion in the primary motor cortex may induce buccofacial apraxia and limb kinetic apraxia as well. Because deglutition contains voluntary and involuntary parts, there is a controversy regarding the application of word “apraxia” for swallowing dysfunction. Notwithstanding, the rippling pattern of swallowing hesitation seems to assume the characteristics of apraxia. Intriguingly, Patient 4 showed the rippling-type swallowing hesitation with a lesion in right primary motor cortex, while Patients 12 and 14, both with lesions including the right primary motor cortex, demonstrated no swallowing hesitation. Thus, the hemisphere dominancy of deglutition might vary among individuals.

### Left Frontal Lesions Responsible for “Stasis” Swallowing Hesitation

On the other hand, the patients with swallowing hesitation characterized by a period of stasis in this study tended to have broad lesions in the left hemisphere. In addition to the primary motor cortex, the orbitofrontal cortex, middle frontal gyrus, inferior frontal gyrus, and insula are entangled at high rates in our patients. For example, Patient 8 (Case 2) showed the typical stasis type of swallowing hesitation due to a broad lesion of the left hemisphere. Lesions in the frontal lobe due to occlusion of the internal carotid artery often become diffuse. In that context, it is noteworthy that Patient 17 (Case 3) with a large part of middle frontal gyrus spared did not show swallowing hesitation, in spite of multiple lesions in the orbitofrontal cortex, inferior frontal gyri, and insula selectively. This indicates us that the lesion of the middle frontal gyrus is important for the establishment of swallowing hesitation, especially the stasis pattern.

The insula, especially its anterior part, receives sensory stimulants in the oral cavity input from nucleus tractus solitarius via the ventroposterior medial nucleus of the thalamus [15]. Further, the anterior part of the insula connects to the primary motor cortex. A lesion of the insula would correlate somewhat with swallowing dysfunction [15] like that seen in this study. The importance of the insula in swallowing is recognized through previous studies, such as a study using functional MRI that demonstrated statistically significant activation in the insula during an oral stereognosis test [16].

**Fig. 2:**
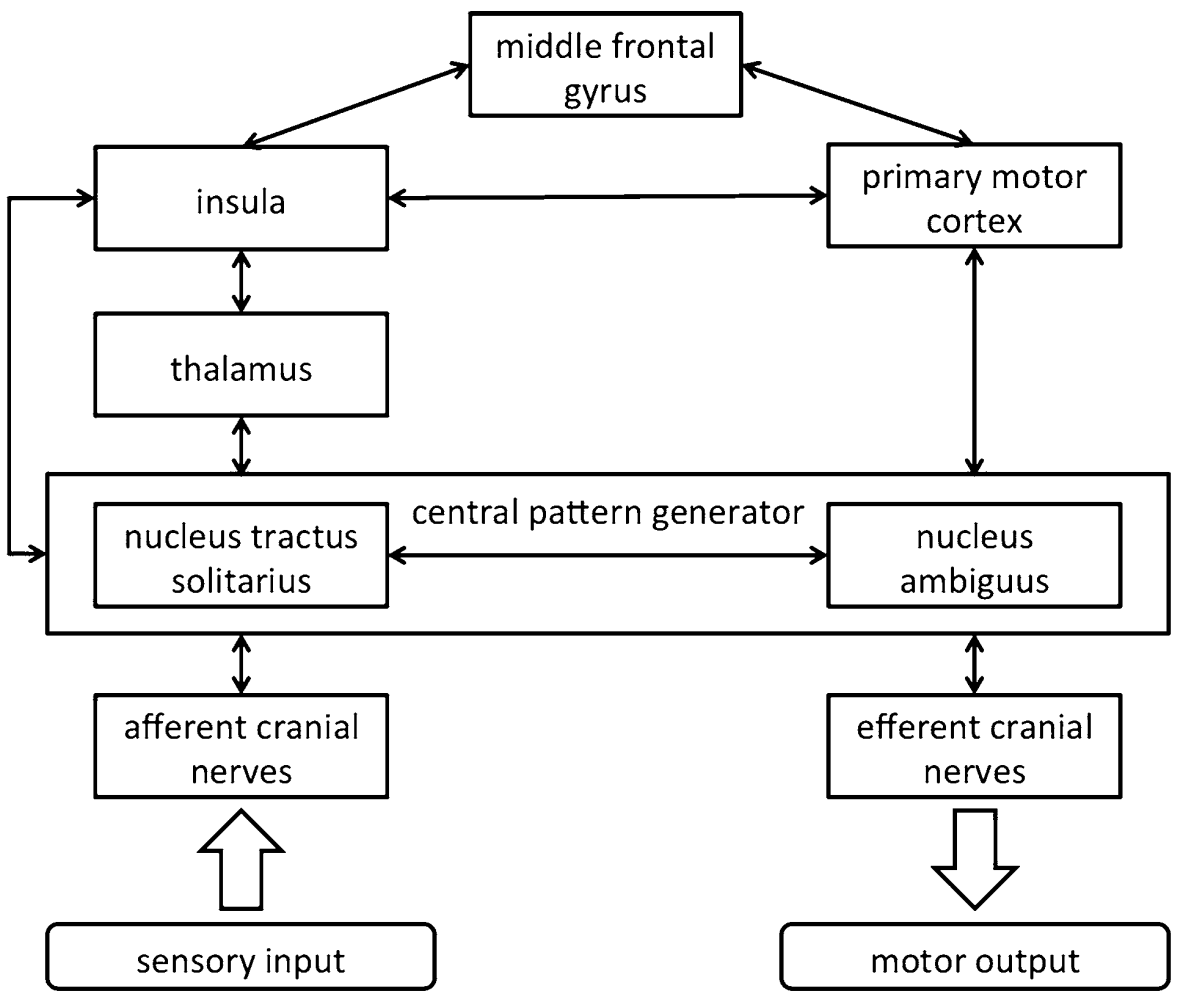
Speculated neural circuit in swallowing. The middle frontal gyrus serves as an association area between the insula as a sensory center and the middle frontal gyrus as a motor center

Although little is known about the relationship between the middle frontal gyrus and deglutition, some researchers have pointed out the importance of the prefrontal cortex, thought to be involved in higher cognition and containing a large part of middle frontal gyrus. The anterior half of the middle frontal gyrus is redefined as the dorsolateral prefrontal cortex (DLPFC) from a functional perspective and investigated for clinical significance. The DLPFC is not only concerned with self-awareness and attentional control but also involved in executive control of working memory. The prefrontal cortex as a frontal association area is also expected to integrate perceived sensory signals with motor commands [17]. An interplay between the insula as a sensory center, primary motor cortex as a motor center, and middle frontal gyrus as an association area of these is essential for proper deglutition. Of course, the medullary central pattern generator (CPG) also plays an important role in the process of voluntary swallowing, such as adjusting the perception of pieces of food by the sensory input coming from the oral cavity without linkage to cortical levels [18]. We speculated on the neural circuit involved in swallowing (Fig. 2).

### Prognosis of Swallowing Hesitation

As mentioned in the previous three case reports, our patients also had good prognosis for swallowing hesitation. Barer reported that nearly 30 % of 357 patients with a unilateral cerebral lesion were initially found to have difficulty swallowing, but most of their symptoms resolved [6]. When we encounter swallowing hesitation during acute management, we should carefully assess the improvement over time. As mentioned above, nuclei of the medulla receive innervation by the bilateral corticobulbar tracts. Therefore, swallowing hesitation caused by a unilateral lesion may often be improved by enhancement and reorganization of the contralateral innervation [19].

### Limitations

This study had several limitations. First, the results of this research have limited effectiveness because of the small number of cases. To determine the association between swallowing hesitation and the responsible lesion with sufficient statistical power, more patients should be studied. Second, the period from onset to VF of some patients was more than 4 weeks, mainly due to their poor general condition in the acute stage. Ideally, we ought to conduct VF within 2 weeks of onset. In order to obtain further information about swallowing hesitation, we should construct a strict protocol for longitudinal follow-up study in liaison with the sub-acute rehabilitation hospital. Third, because this was a retrospective study, we could not compare the results to those of normal age-matched controls. We are organizing a prospective age-matched control study to obtain firm evidence of swallowing hesitation. Fourth, we did not examine the role of oral sensation in swallowing. Because sensory stimulation inside the oral cavity is one of the important triggers for swallowing, it is important to evaluate the oral sensation of patients with dysphagia. We will consider the oral sensation basis of swallowing hesitation, including trigeminal nerve and brainstem, in the future study. In addition, we will evaluate tongue movement qualitatively for detailed clarification of this pathophysiology. In the future, the degree of hesitation also should be evaluated in detail. Finally, we did not perform statistical analysis of the lesion causing swallowing hesitation. Although we attempted to conduct a multivariate analysis, the small number of cases precluded a statistically meaningful result. We recognize the necessity for continuing accumulation of cases.

## Conclusions

To our knowledge, this is the first study to introduce the characteristic and responsible lesions of swallowing hesitation based on multiple cases. Swallowing hesitation caused by acute unilateral infarction was not negligible and could be divided in two different patterns. We propose the possibility that a lesion in the left primary motor cortex is related to the rippling tongue movement type of swallowing hesitation. Moreover, we suggest the possibility that a lesion in the left middle frontal gyrus is linked to the stasis pattern of swallowing hesitation.

## Electronic supplementary material

Below is the link to the electronic supplementary material.

Movie 1 On the sixth day after onset, VF demonstrated swallowing hesitation with a typical repetitive rippling movement of the tongue. Here we show swallowing jelly. (MOV 551 kb)

Movie 2 On the 22nd day after onset, VF demonstrated swallowing hesitation with a typical period of stasis. Here we show swallowing water. (MOV 1413 kb)
